# A rapid on-site analysis method for the simultaneous extraction and determination of Pb^2+^ and Cd^2+^ in cereals†

**DOI:** 10.1039/c9ra05587h

**Published:** 2019-10-15

**Authors:** Ming-hui Zhou, Wei Tian, Jie-qiong Zhang, Xi Chen, Yan-xiang Wu, Song-xue Wang

**Affiliations:** The Academy of National Food and Strategic Reserves Administration (Former Name: Academy of State Administration of Grain, ab. ASAG) No. 11 Baiwanzhuang Str., Xicheng District Beijing 100037 China wsx@chinagrain.org

## Abstract

In order to achieve rapid on-site screening and solve the problem of rapid pretreatment for the determination of lead (Pb^2+^) and cadmium (Cd^2+^) in cereals by a portable electrochemical analyzer with disposable screen-printed electrodes (SPEs), a new reliable and simple extraction method for Pb^2+^ and Cd^2+^ in cereals was developed. The Pb^2+^ and Cd^2+^ in cereals were purified by a mixed solution of 1 mol L^−1^ potassium iodide (KI)/5% vitamin C (VC)/ethyl acetate after being extracted by 10% HNO_3_, which transfers the Pb^2+^ and Cd^2+^ into ethyl acetate after a reaction with KI–VC. Then, the Pb^2+^ and Cd^2+^ were eluted from ethyl acetate with 5% HNO_3_ and were determined by an electrochemical analyzer with screen printed electrodes. Under the optimized conditions, the matrix calibration curves of Pb^2+^ and Cd^2+^ in rice and wheat showed good linear relationships with *R*^2^ > 0.996. The method shows a detection limit (LOD) for Cd^2+^ in rice and wheat of 6.7 μg kg^−1^ and 11.5 μg kg^−1^, and the corresponding values for Pb^2+^ were 34.9 and 31.1 μg kg^−1^, respectively. The relative standard deviation (RSD) was less than 8.7% for Cd^2+^ and Pb^2+^. In addition, the recoveries of the tested reference materials using this method were between 80% and 120%. From sample pretreatment to testing results, the whole process took no more than 25 min, and the operation was simple for operators, green to the environment, cheap in terms of instruments, and above all suitable for on-site detection. The results implied that this portable electrochemical method with new pretreatment may be a good choice for screening Pb^2+^ and Cd^2+^ in cereal samples on-site.

## Abstract 2

To achieve rapid on-site screening rapid pretreatment for the determination of Pb^2+^ and Cd^2+^ in cereals by a portable electrochemical analyzer with disposable screen-printed electrodes, a new reliable and simple extraction method for Pb^2+^ and Cd^2+^ was developed.

## Graphical abstract

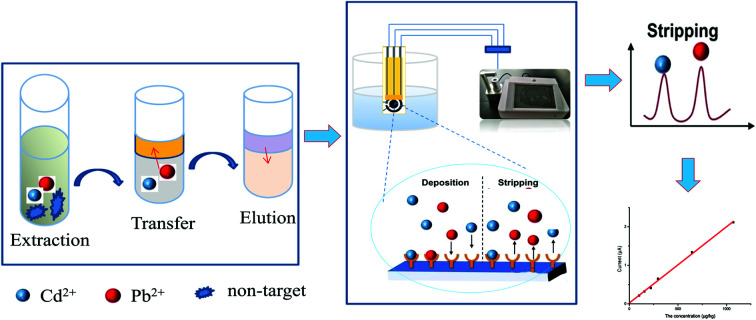

## Introduction

Heavy metals are known to pose a great threat not only to human beings, but also to aquatic plants and animals, due to their high toxicity, high stability and non-biodegradability.^1^ In particular, the two major pollutants Pb^2+^ and Cd^2+^ easily pose an acute or chronic risk even at low concentrations, causing headaches, hypertension, skeletal malformation in fetuses, arthralgia, renal damage and so on.^2,3^ Most of the Pb^2+^ and Cd^2+^ is generated from industry and then released into water and soil, continuously transferred into plants, and accumulated in cereals and humans.^4^ What's more, cereals are the main food crop for half of the world's population.^5^ This mode of diet has greatly increased people's intake of heavy metals and the risk of disease. Therefore, on account of the harmfulness and scattered samples, rapid and on-site detection methods are extremely urgent, and it is of great importance for human health to detect Pb^2+^ and Cd^2+^ in a quick and reliable way.^6^ Especially in countries where rice production has not realized intensive cultivation, such as China, the problem of Pb^2+^ and Cd^2+^ in cereals is salient.

However, there are still some difficulties in realizing the rapid detection of Pb^2+^ and Cd^2+^ in grains on-site. Over the past few decades, there has been increasing interest in the development of detection methods for Pb^2+^ and Cd^2+^. Routine methods for detection are atomic absorption spectroscopy (AAS),^7^ inductively coupled plasma-mass spectrometry (ICP-MS),^8^ inductively coupled plasma atomic emission spectrometry (ICP-AES),^9^ and inductively coupled plasma-optical emission spectrometry (ICP-OES)^10^ and so on. These methods have achieved high precision for detecting heavy metal ions, but they have some disadvantages, such as the high cost of the cumbersome instruments required and their maintenance, the requirement of skilled professionals and being time-consuming.^11,12^ These drawbacks have limited their application in the field. Therefore, it is imperative to establish portable analytical systems.

Electrochemical methods, in particular based on screen printed electrodes (SPEs), are widely used in heavy metal ion detection due to their low cost, simple operation and fast response.^13–15^ Since the 1990s, screen-printed technology has been used for electrochemical sensors,^16^ which are generally used in many fields, especially for water detection. However, they are rarely used in cereal samples because of the complicated matrix interference. Complex matrices of samples, involving proteins, fats, pigments and other ions, can result in a significant gap between the lab value and on-site measurement conditions.^17^ Therefore, complicated matrix interference, low concentrations of heavy metal ions and high levels of electrolyte background are key challenges for the electrochemical measurement performance.^18^ The way to solve these problems partly depends on pretreatment. The traditional digestion method requires a lot of strong acid and it takes a long time (at least 2–3 hours).^19^ Recently, a method which uses diluted acid for extracting Cd^2+^ and Pb^2+^ has been developed for the purpose of abandoning the dangerous strong acid and simplifying the operations.^20–22^ What's more, some researchers have also applied 4-methyl-2-pentanone (MIBK) in pretreatment for further purification. Most of the publications employing MIBK as the solvent extractor focused on pretreatment prior to Cd^2+^ and Pb^2+^ analysis, where it was used widely for water and soil. Compared to traditional pretreatment, this method indeed saved a lot of time, but there are only a few reports on the determination of Pb^2+^ and Cd^2+^ in cereal samples using MIBK extraction methods. Shi *et al.*^23^ used ammonium pyrroline dithiocarbamate (APDC) to coordinate metal ions and then extracted them with MIBK, and the final results complied with the requirements of neutron activation analysis (NAA). Xin *et al.*^24^ used KI to perform a substitution reaction with Pb^2+^ and Cd^2+^ before they were extracted with MIBK. In fact, MIBK is currently a relatively hard-to-get chemical intermediate, and it is considered to be a hazardous air pollutant, especially in high-rise air,^25^ but other common solvents have been rarely studied to replace MIBK. Ethyl acetate is a good alternative to organic solvents because of its non-toxic and excellent properties for extraction. But regarding KI–ethyl acetate extraction for an effective cereal pretreatment method, there has been no report yet.

In this study, we developed a rapid pretreatment approach coupled with an electrochemical method to determine trace Pb^2+^ and Cd^2+^ simultaneously in cereal samples (rice and wheat) with a portable heavy metal screen-printed electrode. In brief, the target Pb^2+^ and Cd^2+^ were simultaneously extracted by diluted acid from cereals, and the ions reacted with organic acids (vitamin C) and potassium iodide to form the associated complexes, which can be transferred to the organic phase and then eluted into inorganic acids. After a simple pretreatment of the samples, the on-site analysis was carried out using disposable screen printed electrodes as the working electrodes. Some of the key factors, such as the extraction rate, elimination of interferences and electrode working conditions, were investigated. Moreover, the analytical performance of this method was validated with certified reference materials in comparison with the ICP-MS method. Under the optimal parameters, the basic standard curve was established. The detection limits, repeatability, and accuracy were all evaluated. In the meantime, real cereal samples collected from markets were used to demonstrate the reliability of the new method in comparison with ICP-MS. The whole detection procedure took less than 25 min, and it supplied a reliable method for the simultaneous detection of Pb^2+^ and Cd^2+^ in cereals on site.

## Experimental

### Chemicals

All certified reference materials (CRMs), namely GBW(E)100350, GBW(E)100348, GBW100361, GBW100378, and GBW100379, were purchased from the National Institute of Metrology, China. ZK012, ZK014, ZK004, ZK001, ZK002, ZK003, FD, FM and other samples containing Cd^2+^ and Pb^2+^ were prepared by the Academy of National Food and Strategic Reserves Administration, China. HNO_3_, ammonium acetate and KI were purchased from Beijing Chemical Reagent Research Institute. VC was purchased from CNW technologies. Ethyl acetate (GR) was purchased from Fisher Chemical. Pb^2+^ and Cd^2+^ standard solutions were purchased from National Institute of Metrology. Electrolyte buffer solutions were kindly provided by Wuhan Zhongkezhikang Biotechnology Co. Ltd.

### Instruments

All solutions were prepared with ultrapure water (resistivity: 18.2 MΩ cm^−1^) from a Milli-Q purification system (USA), and the samples were ground in a grinder (Fritsch, Germany). Cd^2+^ and Pb^2+^ in the extracts was quantified by ICP-MS (7500CX, Agilent, USA). A high-speed centrifuge (3-30KS, Sigma Laborzentrifugen Gmbh, Germany) was used to separate the extracted solutions. The screen printed graphite working electrodes (SPEs) and the electrochemical detector were kindly provided by Wuhan Zhongkezhikang Biotechnology Co. Ltd.

### Cereal sample preparation

In this study, rice and wheat were selected as the representative samples due to them being the most prevalent cereals as a staple food worldwide and the fact that they are commonly regarded as cereals in which heavy metals are easily accumulated. Unlike the simple diluted acid extraction,^26^ the new method could get rid of more soluble impurities. Briefly, the samples were ground into a powder and mixed completely. A portion (0.25 g) was transferred into an extraction tube, and the following solutions were added to the tube in sequence: 10% HNO_3_, 10% VC solution, 2 mol L^−1^ KI solution, and ethyl acetate. The Pb^2+^ and Cd^2+^ were extracted and cleaned from this mixture solution by vigorously vortexing for 10 min on a shaker. After being centrifuged at 4000 rpm for 5 min, the solvent was separated from the aqueous layer. Then, a 1 mL aliquot of the ethyl acetate supernatant was back-extracted with 5% HNO_3_ by hand-shaking for 1 min. After stratification, the organic solvent was abandoned and the aqueous phase was prepared for detection.

### Procedure of the electrochemical method of Pb^2+^ and Cd^2+^

**Fig. 1:**
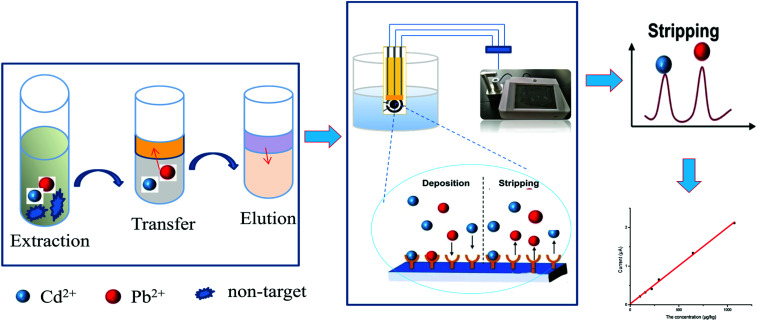
The principle of the portable electrochemical method.

The analysis of Pb^2+^ and Cd^2+^ by the one-strip electrochemical method with solvent extraction was performed in acetate buffer solutions. The SPEs had three electrodes, consisting of a round carbon working electrode (diameter of 2.8 mm), a carbon auxiliary electrode, and a silver pseudo-reference electrode. The working electrode should be activated first. In brief, under stirring conditions, the electrode was immersed in a mixture buffer containing 1 mL of 0.5 mol L^−1^ acetate buffer solution and 40 μL of 1000 mg L^−1^ Hg^+^ standard solution and the deposition potential (−1.3 V) was applied to the working electrode for 200 s. This step was to activate the electrode and form an amalgam on the surface of the electrode, which can improve the sensitivity and stability. After the electrode was activated, 200 μL of sample solution was added to the sample cell that already contained the activated electrode scanned for 200 s and a potential scan was carried out from −1.3 V to 0.6 V. After an equilibration period, the potential scan was carried out for 10 s, and the solution was not stirred in this step. The parameters of the detection conditions were set up, such as frequency (10 Hz), amplitude (25 mV) and scan rate (4 mV s^−1^). All the steps were imposed at room temperature. The main principle and processes are shown in Fig. 1.

### Optimization of the working conditions and matrix-matched calibration curves

In this method, one of the priority issues of the whole detection was the sample extraction procedure, and it was of great importance to extract and concentrate the target metal ions from the cereal samples. In order to obtain a complete extraction rate, the following critical factors were studied, such as the nitric acid concentration, the ratio of sample to diluted-nitric acid (m/v), the extraction pattern and extraction time, and the concentration of KI and VC. Under the optimized working conditions, calibration curves were established. A series of standard cereal materials that contained different concentrations of Pb^2+^ and Cd^2+^ were chosen as the standard matrixes, and calibration curves were evaluated by plotting the current response against concentration. The limit of detection (LOD) (3*σ*/*s*) and the limit of quantification (LOQ) (10*σ*/*s*) were also calculated from the calibrations, where *σ* is the standard deviation of eleven blank measurements and *s* is the slope of the calibration curve.

For optimization of the nitric acid concentration, in this part, 3%, 5%, 10% and 15% HNO_3_ were studied for the extraction rate of Pb^2+^ and Cd^2+^. Different concentrations of acid were added to the certified reference samples, and the operation was carried out according to “*Cereal sample preparation*” and “*Procedure of the electrochemical method of Pb*^*2+*^*and Cd*^*2+*^”. The recoveries of Pb^2+^ and Cd^2+^ were calculated.

For optimization of the ratio of sample to diluted-nitric acid (m/v), the ratios of sample to diluted-nitric acid (m/v) were set as 1 : 5, 1 : 10 and 1 : 15. Through the steps mentioned above in “*Cereal sample preparation*”, the untested liquid was detected. The recoveries of Pb^2+^ and Cd^2+^ were calculated.

For optimization of the extraction time and the extraction method, to improve the extraction rate, the best extraction time and method were evaluated; the extraction time was 3, 5, 10, or 15 min, and at each time, two extraction methods, namely shaking and vortexing, were used to extract Cd^2+^ and Pb^2+^ in both rice and wheat.

For optimization of the concentration of KI, to identify the influence of KI on the method, different concentrations of KI solution (0.1, 0.2, 0.5, 1.0, 2.5, 4.0, 5.0 mol L^−1^) were added during the pretreatment. After testing, the current signals were compared to analyze the influence of KI.

For optimization of the concentration of VC, different concentrations of VC solution (3, 5, 10, 15, and 18%) were added to improve the performance of the method. Then, the trends of the current signals under different conditions were compared to identify the optimum concentration.

For optimization of the concentration of eluent, 5% HNO_3_ and 10% HNO_3_ were used as the primary eluents. The eluted liquid was prepared for detection. The concentration that gave the highest current signal was selected as the appropriate concentration of the eluent.

### Precision and stability

Precision was evaluated by determining the response of seven independent replicates for each sample. Furthermore, in order to perform a comprehensive evaluation, a three-person evaluation model was cited. In this model, three different concentration levels of the sample were used for analysis. The three different concentration levels were selected mainly based on the national standard limit, and high-, medium- and low-contamination samples were selected for analysis. Each person performed the test seven times independently.

### Validation

The applicability of the established method was further investigated with the certified reference materials and some real samples that were collected from different provinces of China. The sample preparation was performed according to the section “*Cereal sample preparation*”, and the experiments were performed by the established method. In the meantime, ICP-MS measurements were carried out following a conventional procedure. Before the measurements, the samples were digested for 50 min by a microwave digestion procedure. And then, the results of the certified reference materials that were obtained by the established method were compared with the reported concentration values and the real sample results that were obtained by ICP-MS were compared with the results that were calculated by the established method.

## Results and discussion

### Optimization of the sample extraction conditions

After the pretreatment of the cereal samples, the supernatant was transferred to the cell to be detected. It was found that the peaks were not smooth and even had a slight offset. That is to say, some organic molecules, such as starch, fats, proteins, *etc.* caused severe interference for determination. In order to obtain a better sensitivity and stability to achieve simultaneous detection of Pb^2+^ and Cd^2+^ in this portable method on-site, some experimental conditions which may affect the extraction and current response were optimized.

### Optimization of the nitric acid concentration

**Fig. 2:**
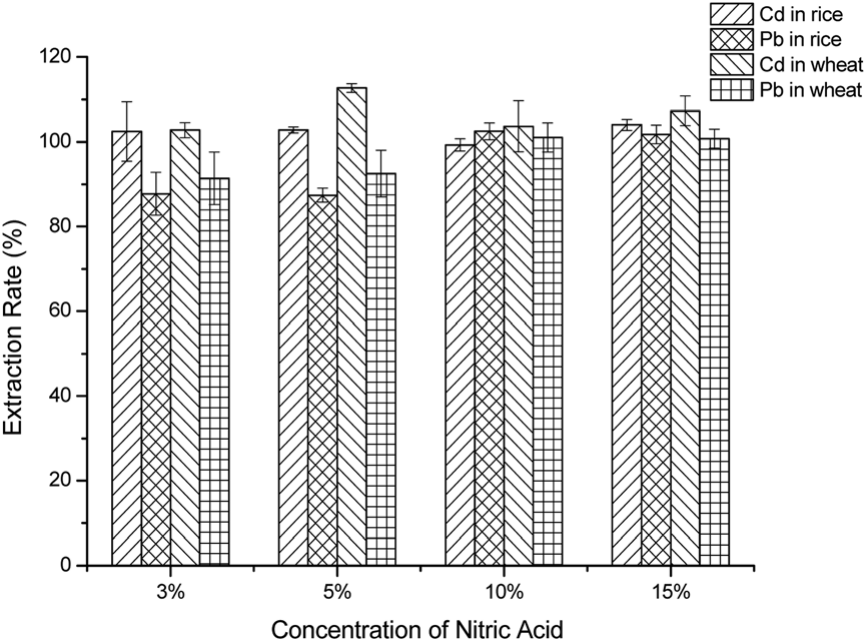
The effect of the concentration of nitric acid.

In order to extract Cd^2+^ and Pb^2+^ simultaneously, the concentration of acid was studied. As the data show, when the acid was at low concentrations, such as 3% HNO_3_ and 5% HNO_3_, the extraction rate of Pb^2+^ was slightly lower than using 10% HNO_3_ (Fig. 2). When the concentration was increased further, the extraction rate of Pb^2+^ decreased gradually. This phenomenon appeared in both rice and wheat. As for Cd^2+^, all concentrations of diluted nitric acid that were used for extracting Cd^2+^ could give a recovery of approximately 100%. That is to say, Cd^2+^ was more easily extracted by dilute acid and was more stable than Pb^2+^ in dilute acid solution. Therefore, considering the extraction rate of Pb^2+^ and the usage of HNO_3_, 10% HNO_3_ was employed in further investigations.

### Optimization of the ratio of sample to diluted-nitric acid (m/v)

**Fig. 3:**
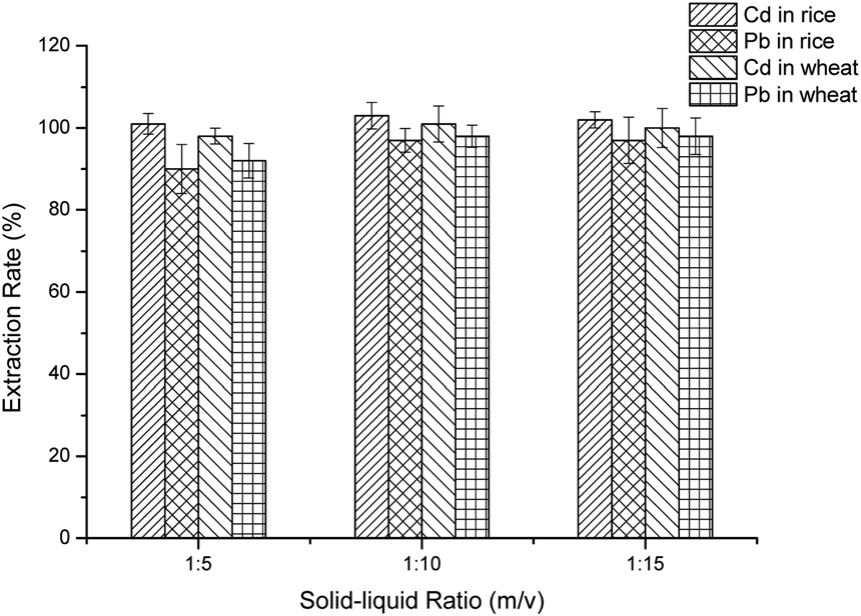
The effect of the solid to liquid ratio for extraction.

The ratio of sample to diluted-nitric acid (m/v) was also an important factor for extraction. Fig. 3 shows the results of the two metal ion recoveries under different ratios of sample mass to diluted-nitric acid volume. At these three ratios, there was no significant change in the extraction rate of Cd^2+^, whether it was rice or wheat. However, the extraction rate of Pb^2+^ rose with an increase in the solid–liquid ratio, and tended to be stable at 1 : 10. The highest recoveries appeared at 1 : 10 for both Pb^2+^ and Cd^2+^. That is to say, at 1 : 10, both Cd^2+^ and Pb^2+^ can achieve the best extraction effect. When the solid–liquid ratio was less than 1 : 5, the mixture was relatively viscous and it was not easy to carry out the following step. Finally, 1 : 10 was chosen for the following experiments.

### Optimization of the extraction time and the extraction modes

**Fig. 4:**
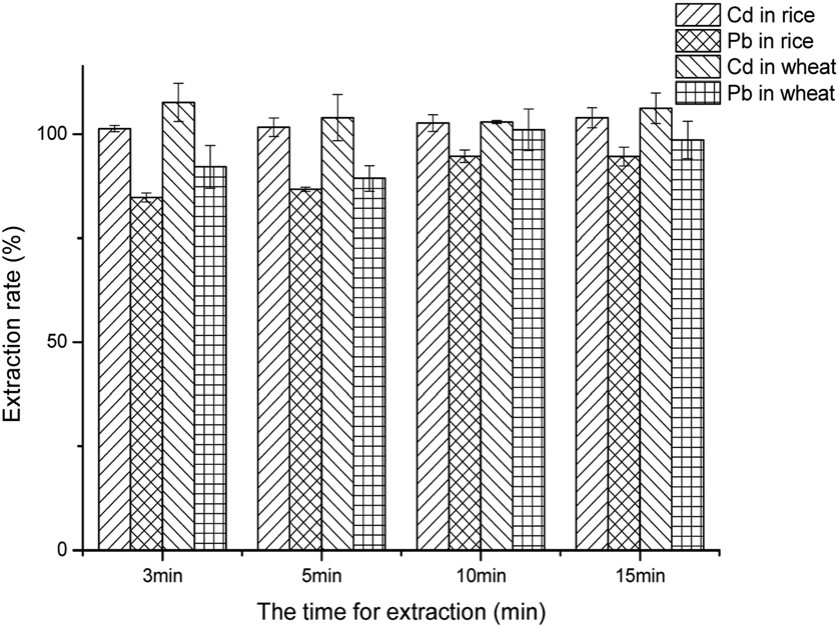
The effect of the extraction time.

Two kinds of extraction modes and their corresponding times were studied. The extraction efficiency of oscillation and vortexing may not be as high as that of ultrasonic extraction, but ultrasonic extraction is not suitable in this method, because it might not be convenient on-site. However, if given more time, the two modes mentioned above may also obtain a good extraction rate. For the extraction time and the extraction mode, no matter whether vortexing or oscillation, the extraction rate of Cd^2+^ in rice and wheat was almost 100% (ESI†), illustrating that Cd^2+^ was very easy to extract in acid solution. This was in good agreement with the previous research.^27^ Unlike Cd^2+^, the extraction rate of Pb^2+^ by the oscillation extraction mode was generally higher than that by vortexing at the same time. It may be the case that the oscillation mode was more intense than the vortexing mode, which led to the sample being in full contact with the extract, so that more Pb^2+^ was brought out. As can be observed in Fig. 4, when the Pb^2+^ in wheat was extracted by vortexing for 10 min, the recoveries of both Pb^2+^ and Cd^2+^ were the closest to 100%. Therefore, these data supplied adequate evidence for a satisfactory extraction rate.

### Optimization of the concentration of KI

**Fig. 5:**
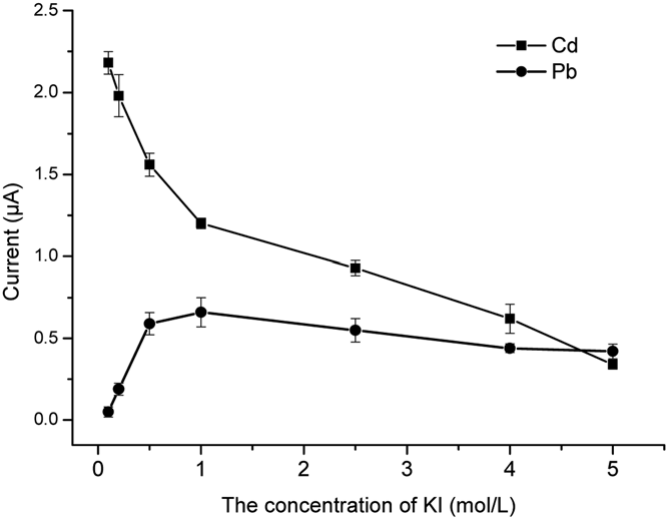
The effect of the concentration of KI.

The main role of the KI is to replace the Pb^2+^ and Cd^2+^ by a displacement reaction, so that the formed Pb^2+^ and Cd^2+^ compounds are more easily dissolved in organic solvents. Some studies have reported that I^−^ can react with Cd^2+^ to form [CdI_4_]^2−^ under acidic conditions and Pb^2+^ can react with I^−^ to form PbI_2_, a yellow precipitate, but this precipitate can be dissolved in excessive KI solution and form complex K_2_[PbI_4_]. The complex formed by KI and heavy metal ions was extracted by the organic phase.^28^ In order to determine whether residual KI could affect the electrochemical current signal, different concentrations of KI solution were added. As can be seen from Fig. 5, when testing the concentration of KI needed for electrochemical detection of Cd^2+^, the signal response exhibited a continuous increase until the concentration reached 1.0 mol L^−1^, and the response value showed no obvious change when the concentration was further increased. In contrast, the signal value of Pb^2+^ declined quickly with increasing concentration of KI until 1.0 mol L^−1^, and then it declined slowly as the concentration of KI kept increasing. As a compromise, 1 mol L^−1^ of KI could be beneficial for the analysis of both Pb^2+^ and Cd^2+^.

### Optimization of the concentration of VC

**Fig. 6:**
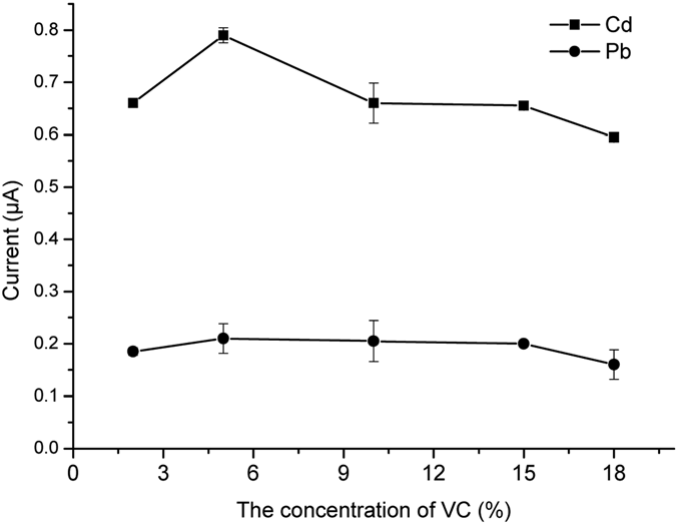
The effect of the concentration of VC.

VC is well known to be a redox-type ion masking agent that can reduce other interfering ions except for Pb^2+^ and Cd^2+^. It is reported that VC can reduce the oxidation of KI in acidic conditions. This study is performed in HNO_3_ and HNO_3_ has certain oxidation ability. Therefore VC is necessary to protect the function of KI. In addition, VC can also reduce some high-valence metal ions to reduce the interference in the determination.^29^ Besides, VC is an organic acid, which is beneficial to the reaction of KI and ions. Therefore, different concentrations of VC solution were added. From Fig. 6, it can be seen that the current response to Cd^2+^ reached the peak when the concentration of VC was 5%. Meanwhile, there was no observable regularity for Pb^2+^. Therefore, 1 mL of 5% VC was used for the subsequent experiments.

### Optimization of the concentration of eluent

For the eluent, the concentration of the back extraction acid was a key point because it can not only affect the elution degree of Pb^2+^ and Cd^2+^ but also affect the pH of the electrochemical buffer to be tested. The influence is indicated in Table 1. The current response with 5% HNO_3_ was much higher than that with 10% HNO_3_ for Cd^2+^, while there were no obvious effects for Pb^2+^. This may be because Cd^2+^ was more sensitive than Pb^2+^ to the pH, and although there was no significant difference in elution rate between 5% HNO_3_ and 10% HNO_3_, the pH of the electrochemical working buffer can be obviously affected. Therefore, 5% HNO_3_ was chosen as the back-extraction eluent.

**Table:** Study on the concentration of eluent

Eluent	The current of rice (μA)		The current of wheat (μA)	
	Cd	Pb	Cd	Pb
5% HNO_3_	1.09	0.36	0.83	0.47
10% HNO_3_	0.62	0.33	0.63	0.47

### Plotting matrix-matched calibration curves

**Fig. 7:**
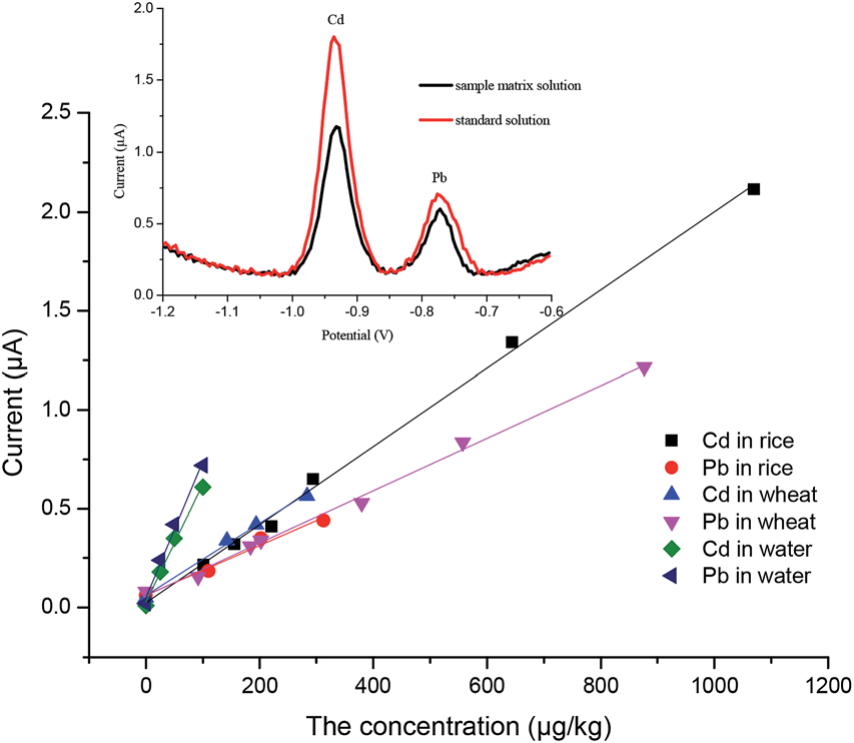
Comparison and analysis of calibration curves of the standard solution and sample solution. Inset: square-wave stripping voltammograms at the SPEs for concentration levels of Cd^2+^ and Pb^2+^ at 12.5 μg L^−1^ and 6.5 μg L^−1^ in 0.5 M acetate buffer; deposition *E*: −1.3 V; deposition time 200 s.

**Fig. 8:**
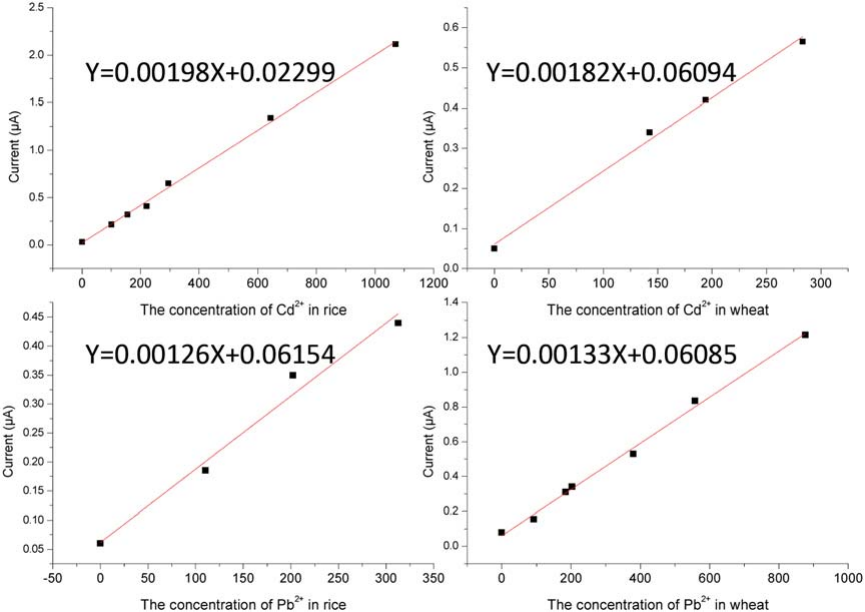
The matrix-matched standard calibration curves of Cd^2+^ and Pb^2+^.

Under the optimum experimental conditions, matrix calibration curves of Pb^2+^ and Cd^2+^ in rice and wheat were obtained with a portable heavy metal electrochemical analyzer. The matrix effect was a ubiquitous phenomenon, and although we had eliminated the matrix effect as far as possible in the pretreatment process, there were still some disturbances left. As shown in Fig. 7, after pretreatment, the spectrum of the sample was almost the same as that of the standard solution, and there were no impurity peaks. However, the current of the sample matrix solution was slightly lower than that of the standard solution at the same concentration. It can also be seen from Fig. 7 that there was no statistical difference in the slope of the same element in the different matrices, except for the standard solutions (*p* < 0.05). More details can be found in the ESI.† The reason for this phenomenon may be the inhibition phenomenon. Reagents have different intensities of inhibition on different target ions. Therefore, the most practical way to solve the matrix effect was the application of matrix-matched standard calibration, which can compensate for the response of the target in the standard solution and the sample solution to the same extent.^30,31^ Considering the above, the external calibration curve method was used and matrix-matched standard calibration curves of rice and wheat were plotted in this work (Fig. 8). For the instrument, we designed different matrix measurement modes, and built in a matrix standard curve corresponding to different matrices. The correlation coefficients were 0.9983 and 0.9980 for Cd^2+^ in rice and wheat, respectively. And the correlation coefficients were 0.9984 and 0.9962 for Pb^2+^ in rice and wheat, respectively. The linearity between the current signal response and the concentration of Cd^2+^ and Pb^2+^ was good in the range of the common concentration range in samples. In practice, the LOD of Cd^2+^ in rice and wheat can be calculated as 6.10 μg kg^−1^ and 9.38 μg kg^−1^, and the LOD of Pb^2+^ in rice and wheat can be calculated as 34.9 μg kg^−1^ and 31.1 μg kg^−1^, respectively. The LOQ of Cd^2+^ in rice and wheat was 20.33 μg kg^−1^ and 31.25 μg kg^−1^, and the LOQ of Pb^2+^ in rice and wheat was 116.4 μg kg^−1^ and 103.7 μg kg^−1^. Compared with the analytical performance of other electrode methods that have been published recently, this developed method showed a consistent sensitivity and low detection limit,^32–34^ with no need for pre-concentration performed using a digestion procedure.

### Precision and stability

Precision was evaluated by determining one sample for seven replicates. RSD was less than 8.7% for Cd^2+^ and Pb^2+^. Furthermore, in the three-person evaluation model, standard materials that contained Cd^2+^ and Pb^2+^ at 100 (±15%) μg kg^−1^, 200 (±15%) μg kg^−1^ and 400 (±15%) μg kg^−1^ were analyzed. Each person tested one sample seven times independently. The results are shown in Table 2 with recoveries between 83% and 120% and RSDs less than 8%. These data indicated that the method had good reproducibility.

The precision and stability of the method for Cd^2+^ and Pb^2+^ in rice and wheat

**Table:** 

	Conc. (μg kg^−1^)	Reviewer 1 (μg kg^−1^)	Reviewer 2 (μg kg^−1^)	Reviewer 3 (μg kg^−1^)	Mean conc. (μg kg^−1^)	RSD (%)	Recovery range (%)		Conc. (μg kg^−1^)	Reviewer 1 (μg kg^−1^)	Reviewer 2 (μg kg^−1^)	Reviewer 3 (μg kg^−1^)	Mean conc. (μg kg^−1^)	RSD (%)	Recovery range (%)
Cd in rice	106	116	124	125	118	7.1	95–120	Cd in wheat	87	99	93	87	90	4.5	93–114
		108	119	126						84	90	93			
		116	113	101						91	90	88			
		124	123	120						95	90	89			
		109	105	128						81	96	93			
		118	116	128						88	90	95			
		104	123	127						89	89	90			
	221	233	232	221	231	4.8	91–113		175	180	178	174	180	5.7	93–114
		218	202	239						163	185	186			
		219	237	239						197	176	176			
		228	238	239						199	168	179			
		241	224	230						182	171	176			
		223	246	250						189	163	182			
		224	229	241						200	181	179			
	442	369	416	441	450	6.9	83–113		400	382	383	390	398	4.5	88–106
		420	474	424						378	379	399			
		454	419	473						425	396	408			
		446	423	469						418	416	405			
		447	462	465						387	389	393			
		491	491	452						418	407	403			
		498	450	472						352	415	413

**Table:** 

	Conc. (μg kg^−1^)	Reviewer 1 (μg kg^−1^)	Reviewer 2 (μg kg^−1^)	Reviewer 3 (μg kg^−1^)	Mean conc. (μg kg^−1^)	RSD (%)	Recovery range (%)		Conc. (μg kg^−1^)	Reviewer 1 (μg kg^−1^)	Reviewer 2 (μg kg^−1^)	Reviewer 3 (μg kg^−1^)	Mean conc. (μg kg^−1^)	RSD (%)	Recovery range (%)
Pb in rice	101	97	99	119	106	6.9	93–114	Pb in wheat	92	92	107	89	96	5.4	95–112
		101	94	114						100	95	96			
		94	101	114						97	88	97			
		104	100	111						100	91	93			
		107	98	115						88	97	96			
		110	106	108						101	103	100			
		106	108	115						92	93	103			
	202	188	216	224	221	7.6	93–120		184	167	175	164	180	5.0	89–105
		223	201	222						177	186	190			
		222	189	238						189	188	186			
		202	214	242						192	171	184			
		241	242	213						183	170	185			
		222	242	235						179	193	168			
		232	230	208						183	168	175			
	405	481	433	466	446	5.6	100–120		368	365	339	364	358	5.3	90–107
		505	424	439						370	333	347			
		468	463	450						355	335	392			
		427	496	411						379	335	365			
		470	445	415						352	343	393			
		454	412	434						368	339	371			
		440	407	455						368	330	373			

### Application to analysis of cereal samples

The practical applicability of the established method was further investigated with a real sample test. Sample preparation was performed according to the “*Cereal sample preparation*” section, and the following experiment was performed by the established method. The results were compared with ICP-MS to obtain a relative recovery. Table 3 shows satisfactory recovery results of 80–120% for both Pb^2+^ and Cd^2+^, and the results confirmed that the developed method has good availability.

**Table:** Comparison of the results of the electrochemical method and ICP-MS for real samples

Sample	Cd			Pb		
	ICP-MS (μg kg^−1^)	Electrochemical method (μg kg^−1^)	Recovery (%)	ICP-MS (μg kg^−1^)	Electrochemical method (μg kg^−1^)	Recovery (%)
Rice 1	261	248	95.02	220	251	114.09
Rice 2	343	286	83.38	110	100	90.91
Rice 3	611	586	95.91	297	240	80.81
Rice 4	169	153	90.53	—	—	—
Rice 5	240	212	88.33	—	—	—
Rice 6	—	—	—	340	335	98.53
Wheat 1	80	96	120.00	410	384	93.66
Wheat 2	155	166	107.10	220	178	80.91
Wheat 3	211	253	119.91	209	187	89.47
Wheat 4	575	623	108.35	402	393	97.76
Wheat 5	—	—	—	100	84	84.00

## Conclusions

In summary, a rapid method for the detection and determination of Cd^2+^ and Pb^2+^ in rice and wheat based on electrochemical analysis with disposable screen-printed electrodes was established. The important sample pretreatment for the extraction and purification of the targeted ions was optimized and developed. With the optimal conditions, the electrochemical platform exhibited acceptable stability and reproducibility, and good portability. The present study demonstrated the excellent and practical potential of this method for the simultaneous determination of Cd^2+^ and Pb^2+^*in situ*, with the obvious advantages of simplicity, high speed, and high sensitivity. Moreover, the whole process of high-throughput analysis could be completed in 25 min with a relatively low cost, which is especially suitable for high-throughput analysis. The electrochemical platform also exhibited acceptable stability and reproducibility, and good portability, which are of great importance for screening Pb^2+^ and Cd^2+^ in cereals on-site. Therefore, the method that we have developed is acceptable to monitor Pb^2+^ and Cd^2+^ simultaneously in cereal samples on-site.

## Conflicts of interest

There are no conflicts to declare.

## Supplementary Material

RA-009-C9RA05587H-s001
