# Supplementary material for: A rapid on-site analysis method for the simultaneous extraction and determination of Pb2+ and Cd2+ in cereals
Source: RSC Adv. 2019 Oct 15;9(56):32839–47. doi: 10.1039/c9ra05587h (PMC9073185; doi:10.1039/c9ra05587h)
Supplement: RA-009-C9RA05587H-s001 [file RA-009-C9RA05587H-s001.pdf]

Electronic Supplementary Material (ESI) for RSC Advances.
This journal is © The Royal Society of Chemistry 2019

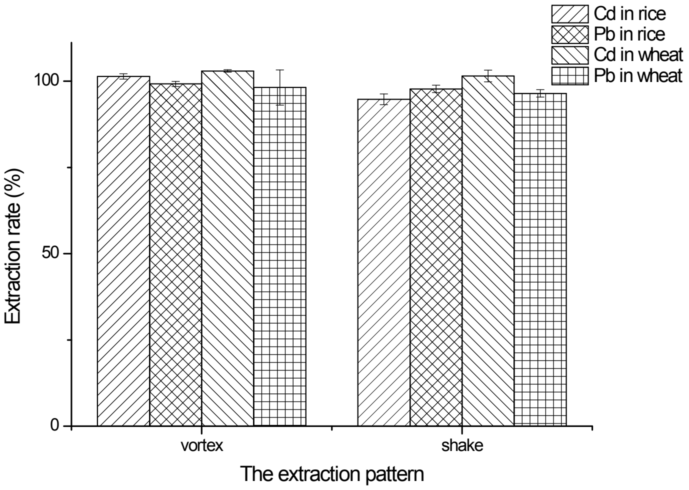

Fig.1 The effect of the extraction pattern

Table 1 Significant analysis of slopes of curves

<table>
<tr><th></th><th colspan="2">Cd^{2+}</th><th colspan="2">Pb^{2+}</th></tr>
<tr><td>Rice</td><td rowspan="2">P=0.358*</td><td rowspan="3">P=0</td><td rowspan="2">P=0.623*</td><td rowspan="3">P=0</td></tr>
<tr><td>Wheat</td></tr>
<tr><td>Water</td><td></td><td></td></tr>
</table>

Note:*P>0.05, no significant difference
